# Reaction-Diffusion Pattern in Shoot Apical Meristem of Plants

**DOI:** 10.1371/journal.pone.0018243

**Published:** 2011-03-29

**Authors:** Hironori Fujita, Koichi Toyokura, Kiyotaka Okada, Masayoshi Kawaguchi

**Affiliations:** 1 Division of Symbiotic Systems, National Institute for Basic Biology, National Institute for Natural Sciences, Okazaki, Japan; 2 Laboratory of Plant Organ Development, National Institute for Basic Biology, National Institute for Natural Sciences, Okazaki, Japan; 3 Department of Botany, Graduate School of Science, Kyoto University, Kyoto, Japan; 4 Department of Basic Biology, School of Life Science, Graduate University for Advanced Studies (SOKENDAI), Okazaki, Japan; Lund University, Sweden

## Abstract

A fundamental question in developmental biology is how spatial patterns are self-organized from homogeneous structures. In 1952, Turing proposed the reaction-diffusion model in order to explain this issue. Experimental evidence of reaction-diffusion patterns in living organisms was first provided by the pigmentation pattern on the skin of fishes in 1995. However, whether or not this mechanism plays an essential role in developmental events of living organisms remains elusive. Here we show that a reaction-diffusion model can successfully explain the shoot apical meristem (SAM) development of plants. SAM of plants resides in the top of each shoot and consists of a central zone (CZ) and a surrounding peripheral zone (PZ). SAM contains stem cells and continuously produces new organs throughout the lifespan. Molecular genetic studies using *Arabidopsis thaliana* revealed that the formation and maintenance of the SAM are essentially regulated by the feedback interaction between WUSHCEL (WUS) and CLAVATA (CLV). We developed a mathematical model of the SAM based on a reaction-diffusion dynamics of the WUS-CLV interaction, incorporating cell division and the spatial restriction of the dynamics. Our model explains the various SAM patterns observed in plants, for example, homeostatic control of SAM size in the wild type, enlarged or fasciated SAM in *clv* mutants, and initiation of ectopic secondary meristems from an initial flattened SAM in *wus* mutant. In addition, the model is supported by comparing its prediction with the expression pattern of *WUS* in the *wus* mutant. Furthermore, the model can account for many experimental results including reorganization processes caused by the CZ ablation and by incision through the meristem center. We thus conclude that the reaction-diffusion dynamics is probably indispensable for the SAM development of plants.

## Introduction

A major subject of developmental biology is how stationary patterns are generated from homogeneous fields. In 1952, in order to account for this issue, Turing proposed the reaction-diffusion model in which stable patterns are self-organized by diffusible components interacting with each other [1]. Whereas this Turing model has been extensively studied by mathematical biologists [2]–[4], until recently it has not been widely accepted by experimental biologists. However, following the description in 1995 of a Turing pattern in the skin pigmentation of marine angelfish [5], the Turing model has attracted attention from developmental and molecular biologists. However, as most of morphogenetic events of animals are irreversible, the patterns that we can observe have been completed and are fixed. Therefore, it would be difficult to verify whether or not the reaction-diffusion pattern plays essential roles in morphogenesis processes in animals [6], [7].

The shoot apical meristem (SAM) of plants resides in the top of the shoot and repetitively produces leaves, branches, and flowers. Whereas many morphogenetic events in animals are completed during embryogenesis, SAM continuously forms new organs throughout the lifespan. SAM is spatially restricted to a small area with an almost constant cell population despite active cell division. The SAM consists of a central zone (CZ) and a surrounding peripheral zone (PZ), which are distinct from an outer differentiated region named the organ zone (OZ) [8]. Stem cells in the SAM are located in the outermost cell layers of the CZ region, and are positively controlled by a group of cells, termed the organizing center (OC), located beneath the stem cells. Many genes show variable levels of expression in different zones of the SAM [9]. Molecular genetic studies in *Arabidopsis thaliana* revealed that many genes are involved in SAM formation and that a feedback interplay between *WUSCHEL* (*WUS*) and *CLAVATA* (*CLV*) is central to the regulation of the SAM [10]–[13]. Mutation of *WUS* or *CLV* results in opposite phenotypes: *clv* mutants have enlarged meristems and frequently generate fasciated and bifurcated shoots [14]–[16]; *wus* mutants initially form a flat shoot apex without leaf primordia, in contrast to the dome-shaped structure of the wild type, suggesting that WUS is a positive regulator of SAM [17]. Interestingly, the *wus* mutant initiates ectopic secondary shoot meristems across the flattened apex, resulting in the formation of a bushy plant with a number of shoots and leaves. It is unclear why and how weakened WUS activity in the SAM leads to the production of so many ectopic meristems. A small peptide derived from CLV3 is perceived as a ligand by the leucine-rich repeat receptor-like kinase CLV1, and possibly by CLV2-CORYNE (CRN) complex and RECEPTOR-LIKE PROTEIN KINASE 2 (RPK2) to restrict *WUS* expression [18]–[22]. In contrast, the homeodomain transcription factor WUS promotes the *CLV3* expression in a non-cell-autonomous manner [19], [24], and also activates its own expression [25], [26].

To date, three mathematical models for the SAM pattern formation using reaction-diffusion system have been proposed based on WUS-CLV dynamics [27]–[29]. These models can explain autonomous pattern formation in the SAM, for example, the expression of *WUS* is stably established at the meristem center in the wild type, is enlarged by defects of *CLV*, and is regenerated following CZ ablation. However, these models do not take into account effects of cell division and spatial restrictions of the meristem and, accordingly, cannot explain the derivation of morphological features such as homeostatic control despite cell proliferation in the wild type and drastic morphological changes in *clv* and *wus* mutants. Therefore, we developed an alternative mathematical model to describe the mechanism underlying SAM proliferation and patterning by integrating cell division and spatial restrictions of the meristem into the reaction-diffusion dynamics based on WUS-CLV regulation.

## Results

### Basic Model for SAM Dynamics

We developed as simple a mathematical model as possible because we aimed to understand the essential dynamics that underlie the proliferation and patterning of the SAM in plants. Our SAM model is based on WUS-CLV dynamics and the spatial restrictions of these dynamic interactions.

#### (I) WUS-CLV dynamics

**Figure 1:**
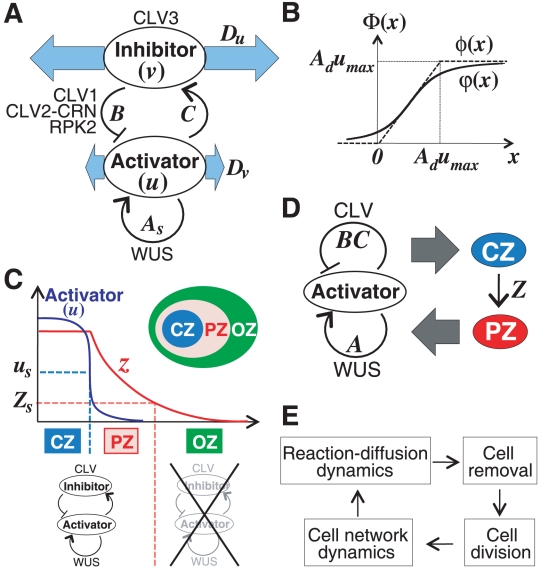
Model framework. (A) Schematic representation of the WUS-CLV dynamics with respect to the activator-inhibitor system. (B) 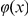 (solid line, Equation (2a); *n* = 2.0) and 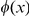 (dashed line, Equation (2b)) are constraint functions ranged between 0 and 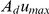. (C) Schematic representation of a spatially restricted SAM. While 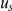 is the threshold of the activator (blue line) for the CZ differentiation, *Z_s_* is the threshold of diffusible molecule *z* (red line) for the SAM differentiation. (D) The SAM is regulated by the interaction between WUS-CLV dynamics at the molecular level and CZ-PZ relationship at the tissue level. (E) The procedure of numerical calculations is divided into four steps (for details, see Materials and Methods).

**Figure 2:**
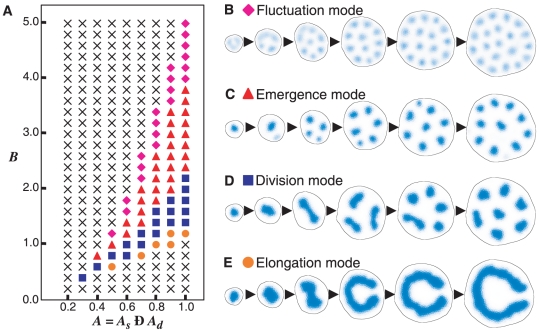
Stem cell proliferation modes. (A) The proliferation mode clearly depends on parameters 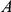 and 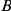. (B–E) Pattern evolution of the reaction-diffusion system in relation to cell division is classified into four proliferation groups: the fluctuation mode (B), emergence mode (C), division mode (D), and elongation mode (E). See also Movies S1, S2, S3, S4 (B–E, respectively). The intensity of the blue indicates the activator concentration (B–E).

Pattern formation by a Turing system has been extensively studied, especially the activator-inhibitor system. In this system, the activator enhances its own production and also production of the inhibitor, while the inhibitor represses activator synthesis [2]–[4]. Here, we modeled WUS-CLV dynamics by reference to the activator-inhibitor system, because the two systems have very similar regulatory interactions (Fig. 1A). Thus, in our model, WUS and CLV equate to the activator and inhibitor, respectively. In more detail, the diffusible peptide CLV3 corresponds to the inhibitor, and CLV1, CLV2-CRN, and RPK2 are involved in its downstream pathway for repressing the activator. On the other hand, as it is not known whether WUS moves between cells, we assume that WUS is involved in the self-activation pathway of the activator, a hypothetical diffusible molecule distinct from WUS in the model. It should be noted that the model has two distinct feedback loops centering on the activator: the positive feedback loop depending on WUS and the negative feedback loop via CLV signaling (Fig. 1D). The basic dynamics of the activator (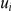) and inhibitor (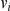) in the *i*-th cell is described by the following form of equations:
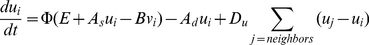
(1a)

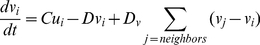
(1b)with the constraint condition in the activator synthesis (Fig. 1B),
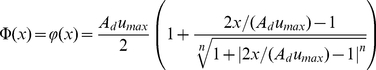
(2a)or
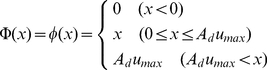
(2b)where 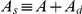, 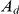, 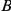, 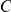, 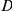, 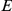, 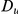, 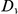, 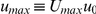, and 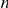 are positive constants, and 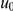 is the equilibrium value of the activator (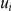) in a simplified form by Equations (3) without space. 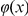 is a sigmoidal function ranged between 0 and 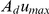 (Fig. 1B). The constraint on the activator synthesis 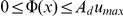 results in that on the activator concentration 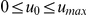, because the equilibrium condition in Equation (1a) without space leads to the equation 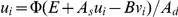. Three terms of the right hand side of Equation (1a) or (1b) represent the synthesis, degradation, and diffusion of the activator or inhibitor, respectively. That is, the activator is induced by itself in the strength 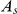, is repressed by the inhibitor in the intensity of 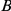, decays at the rate 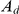, and diffuses between adjacent cells with the diffusion coefficient 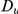. On the other hand, the inhibitor is induced by the activator in the strength 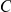, decays at the rate *D*, and diffuses with the diffusion coefficient 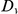. Therefore, the functional strength of WUS is represented by 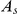 in the model, because the activator and WUS positively regulate each other, in other words, the activator is self-induced via WUS (Fig. 1A). On the other hand, mutations in *CLV* can result in the change of 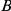 or 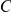.

We also examined a simplified version written by
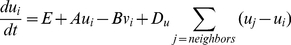
(3a)

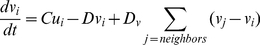
(3b)with the constraint condition of 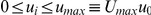; 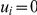 if 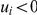 and 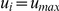 if 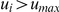. Equation (3a) can be obtained from Equation (1a) by linearizing the activator synthesis and imposing the constraint condition on the activator concentration instead of its synthesis. A theoretical analysis indicates that the positive and negative feedback strengths are associated with 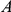 and 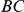, respectively, in this simplified dynamics (Fig. 1D, see Methods S1).

#### (II) Spatial restrictions on WUS-CLV dynamics

The SAM in plants is usually limited in its size, indicating that there must be a mechanism for its spatial restriction. In *A. thaliana*, the CZ is known to induce formation of the PZ, but the detailed molecular mechanism is still unclear [11], [30]. In order to incorporate this feature in our model, we have assumed that a diffusible factor (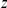) is present. The CZ is defined as the cells in which the activator is expressed at levels greater than the threshold concentration of 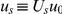 (Fig. 1C, blue broken line). In CZ cells, the diffusible factor *z* is synthesized at the rate of 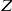; diffusion of the factor generates a gradient (Fig. 1C, red solid line) and induces formation of the PZ. The PZ and OZ are differentiated by having a *z* concentration higher or lower, respectively, than a fixed threshold value (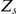) (Fig. 1C, red broken line). Accordingly, parameter 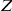 represents the strength of PZ induction (Fig. 1D). The dynamic interaction of WUS and CLV is spatially restricted to the CZ and PZ and does not occur in the OZ, by limiting the activator synthesis to the CZ and PZ. Thus, in PZ cells, the activator is synthesized but remains at very low levels (Fig. 1C). The basic dynamics including the spatial restriction is described by the following equations:
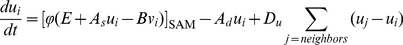
(4a)

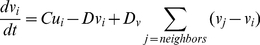
(4b)

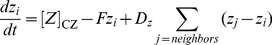
(4c)with
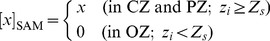
(5a)

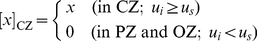
(5b)where 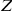, 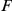, 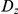, 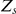, and 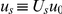 are positive constants. Note that, in this model, the SAM is controlled by the interaction between two regulations: WUS-CLV dynamics at the molecular level and CZ-PZ relationship at the tissue level (Fig. 1D). That is, WUS-CLV dynamics induces the CZ and subsequent PZ, and in turn the SAM spatially defines WUS-CLV activity.

The numerical simulations were performed by a repeated sequence of all or subsets of the four steps: cell network dynamics, reaction-diffusion dynamics, cell removal, and cell division (Fig. 1E). In the steps of the cell network dynamics and reaction-diffusion dynamics, numerical calculations were carried out until an almost steady state. Detailed conditions and parameter values of each numerical analysis are described in Material and Methods, Methods S1, and Table S1.

### Effect of Expressional Separation Between *WUS* and *CLV3*

The expression pattern of WUS-CLV dynamics is spatially regulated in a two-dimensional manner, because its expression domain changes drastically in the lateral direction by defects in the dynamics but does not longitudinally across cell layers [18]–[20], [23], [30]. We therefore modeled SAM pattern formation in two-dimensional space. *CLV3* is, however, exclusively expressed in the outermost cell layers, while *WUS* is expressed in the underlying layers [10]–[13], [18], [24]. We examined the effect of this expressional separation using a simplified two-layered cell network. This analysis indicated that while stable patterns develop in the absence of any expressional restrictions (Fig. S1A), they are completely disrupted by introducing expressional separation in which the activator and inhibitor are synthesized only in the lower or upper layers, respectively (Fig. S1B). We presume that this disappearance of patterning results from the retardation of signal transition from the activator to the inhibitor, because activator synthesized in the lower layer cannot induce the inhibitor until after reaching the upper layer. In fact, stable patterns are restored by adding another diffusible factor (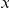) into the signaling pathway from the activator to inhibitor (Fig. S1C). Furthermore, the patterns depended on the diffusion coefficient of this factor (Fig. S1D). That is, pattern restoration requires that the diffusion coefficient of 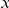 (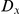) is sufficiently larger than that of the activator (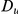) (Fig. S1D). This evidence suggests that the *CLV3* induction pathway involves an unknown diffusible signal molecule other than the activator. These results indicate that pattern formation caused by WUS-CLV dynamics in the SAM is essentially governed by the activator-inhibitor mechanism. Therefore, for simplification, we performed the numerical analyses described below using a conventional activator-inhibitor system in Fig. 1A, with single-layered cell networks.

### Stem Cell Proliferation Mode

#### (I) Pattern evolution caused by area expansion

Since the SAM has the potential for continuous growth due to active cell division, we first investigated the effect of cell division in the absence of any spatial restrictions. This analysis showed that pattern evolution could be classified into four modes:

- **Elongation mode**: an initial spot with high activator concentrations continues to elongate to form stripes as the meristem grows (Fig. 2E and Movie S4).
- **Division mode**: spots continue to multiply by binary fission after their elongation (Fig. 2D and Movie S3).
- **Emergence mode**: spots multiply by the appearance of new spots from areas free of these (Fig. 2C and Movie S2). These three modes generate stable patterns with strong expression of the activator.
- **Fluctuation mode**: spots with weak activator levels continuously move and also elongate, increase by division, and merge with neighboring spots (Fig. 2B and Movie S1).

These proliferation patterns during area expansion are similar to those identified in previous numerical studies [4], [31], [32]. Since the region with high activator concentrations corresponds to the CZ or the stem cells, the proliferation mode represents the growth pattern of stem cells in the absence of spatial restrictions. The proliferation mode is affected by the dynamic balance between the positive and negative feedback loops. Thus, the mode shifts sequentially from elongation, to division, then to emergence, and finally to the fluctuation mode as the negative feedback increases in strength compared with the positive feedback by decreasing 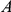 or increasing 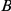 (Fig. 2A). The effect of 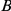 on the proliferation mode is the same as that of 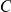 (compare with Fig. S2). This fact is consistent with a theoretical analysis (see Methods S1). In addition, a similar effect on the proliferation mode is obtained in the simplified dynamics expressed by Equations (3) (Fig. S3). This result indicates that the proliferation mode is not qualitatively affected by the nonlinearity of the dynamics.

#### (II) Effect of constraint condition of the dynamics

It is known that the constraint condition has a crucial effect on pattern formation in reaction-diffusion systems. For example, while the activator-inhibitor system can generate spotted, striped, or reverse spotted patterns on a fixed two-dimensional plane [2]–[4], [33], these patterns are responsive to the ratio of distances from the equilibrium to the upper and lower limitations of the activator [33]. That is, the spotted pattern, the reverse spotted pattern, and the striped pattern are generated when the equilibrium is closer to the lower limitation, or closer to the upper limitation, or equally distant from the both limitations, respectively.

Our model dynamics explicitly includes the constraint condition of the activator, and it is shown that this constraint has crucial effects on the proliferation mode during cell division (Fig. S4A). When the equilibrium of the activator is situated at the exact middle between the upper and lower limitations (Fig. S4A, 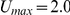), regions with high and low activator concentrations cover almost equivalent areas, resulting in the stripe mode (Fig. S4B). As the upper limitation becomes high by increasing 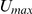, the region with high concentrations becomes small compared to that with low concentrations, and accordingly becomes to generate spots rather than stripes. Resultantly the pattern shifts to the elongation mode, division mode, fluctuation mode, and emergence mode (Fig. S4A, 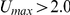). In contrast, as the upper limitation becomes low by decreasing 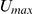, the pattern shifts to the reverse elongation mode (Fig. S4C), reverse division mode (Fig. S4D), reverse fluctuation mode (Fig. S4E), and reverse emergence mode (Fig. S4F). In these cases, spots with low concentrations grow according to each proliferation mode. The SAM of plants probably has the condition that can generate the spotted pattern, because the expression of WUS-CLV system usually results in a spot-like appearance. Therefore, we used a large value of 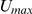 in the numerical simulations in this article.

### SAM Patterns Generated by the Model

**Figure 3:**
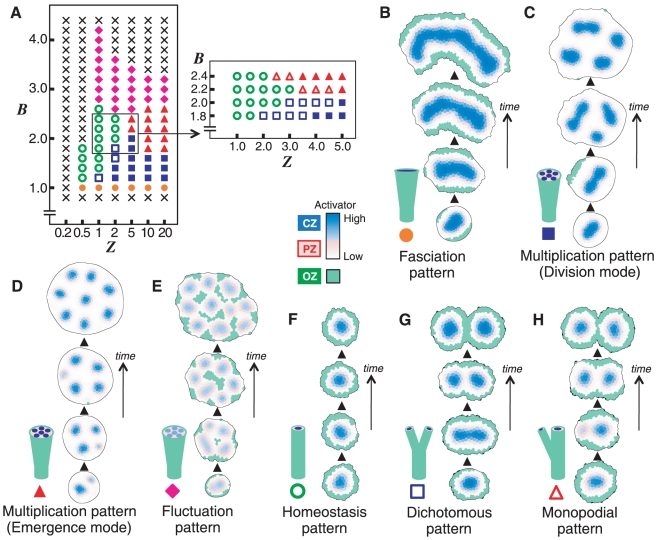
SAM patterns generated by the model. (A) The model generates different SAM patterns by varying 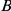 (WUS-CLV dynamics) and 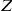 (the spatial restriction). Crosses indicate situations where no patterns are generated. (B–H) SAM patterns can be divided into six groups according to their structure and proliferation mode. See also Movies S5, S6, S7, S8, S9, S10, S11 (B–H, respectively). Blue and red indicate the activator concentration in the CZ and PZ, respectively. The green area indicates the OZ.

The SAM in plants usually does not proliferate indefinitely but is strongly limited to a small area. Accordingly, we investigated the effect of spatial restriction. In this analysis, the SAM patterns that developed from an initial CZ spot are divided into six groups according to their structure and proliferation (Fig. 3).

#### (I) Runaway proliferation

When there is strong induction of the PZ due to a large *Z* component, the resulting patterns of proliferation produce an enlarged SAM with spots or stripes of the CZ. **(i)** The **fasciation pattern** produces an enlarged SAM with a strikingly elongated CZ by the elongation mode (Fig. 3B and Movies S5). **(ii)** The **multiplication pattern** generates multiple CZ spots by the division mode (Fig. 3C and Movie S6) or by the emergence mode (Fig. 3D and Movie S7). **(iii)** The **fluctuation pattern** forms multiple weak CZ spots that proliferate by the fluctuation mode (Fig. 3E and Movie S8). In these patterns, runaway proliferation of stem cells is caused by a chain reaction between PZ expansion and CZ growth.

#### (II) SAM breakdown

In contrast to runaway proliferation, when there is weak PZ induction due to a small *Z*, the PZ area induced by the CZ is too small to maintain the CZ cells, leading to the disappearance of the CZ and subsequent breakdown of the SAM (Fig. 3A, 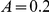).

#### (III) Homeostasis pattern

Under the intermediate condition between runaway proliferation and SAM breakdown, **(iv)** a **homeostasis pattern** appears in which the SAM keeps an almost constant cell population with a single CZ spot at its center (Fig. 3F and Movie S9). This results from a balance between cell multiplication by division and cell loss from the SAM. In other words, through the homeostasis pattern, the plant prevents runaway proliferation of the stem cells by constricting the size of the meristem. We named this effect “stem cell containment”. Whether or not containment occurs will depend on the proliferation mode: containment readily occurs in the division and emergence modes, but is difficult in the elongation and fluctuation modes (Fig. 3A).

#### (IV) Branching-related patterns

The intermediate condition between the homeostasis pattern and runaway proliferation produces patterns related to shoot branching, in which each CZ spot develops into a separate independent SAM (Fig. 3A). These patterns fall into two classes according to their proliferation mode: **(v) dichotomous pattern** by the division mode (Fig. 3G and Movie S10) and **(vi) monopodial pattern** by the emergence mode (Fig. 3H and Movie S11). The two patterns respectively resemble dichotomous branching and monopodial branching in plant shoots.

#### (V) Effect of relative frequency of cell division between CZ, PZ, and OZ

It is known that cell division rate in the SAM is distinct between the CZ and PZ, that is, the PZ shows a more rapid rate of cell division than the CZ [34], [35]. Thus we investigated the effect of relative frequency of cell division on the SAM pattern formation. A variety of relative frequencies does not affect the homeostasis pattern formation, with the exception that extremely high division rates in the PZ compared to the CZ generate the dichotomous pattern (Fig. S5). This result suggests that spatial heterogeneity of cell division activity does not have a large effect on the SAM patterning.

### Regulation of SAM Patterns in Plants

**Figure 4:**
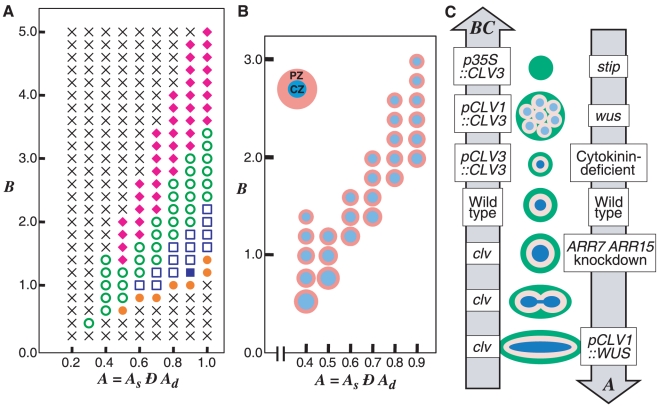
Effect of WUS and CLV on SAM patterning. (A) As 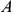 increases or 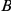 decreases, the SAM pattern shifts sequentially from the fluctuation pattern (filled diamonds), to the homeostasis pattern (open circles), then to the dichotomous pattern (open squares), and finally to the fasciation pattern (filled circles). Crosses indicate situations where no patterns are generated, and filled square indicates the multiplication pattern by the division mode. (B) The SAM area in the homeostasis pattern expands as 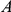 increases or 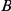 decreases. The blue and red areas indicate the relative sizes of the CZ and PZ, respectively. (C) The effect of 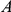 and 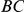 on the SAM patterning is summarized schematically. The predictions of our model agree with many experimental results (for details, see text). The blue, red, and green areas indicate the CZ, PZ, and OZ, respectively.

SAM patterning with regard to the *WUS* and *CLV* genes (which are associated with 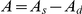 and 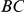, respectively, in our model) has been intensively studied in *A. thaliana* [10]–[13]. Thus, the effect of 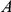, 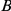 and 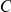 was investigated in detail under an intermediate containment condition that induces the homeostasis pattern. As 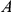 increases or 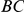 is reduced, the SAM pattern shifts from SAM breakdown, to the fluctuation pattern, then to the homeostasis pattern, then to the dichotomous pattern and finally to the fasciation pattern (Fig. 4A and Fig. S6). That is, 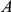 and 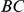 parameters have opposite effects (Fig. 4C).

#### (I) Wild type

**Figure 5:**
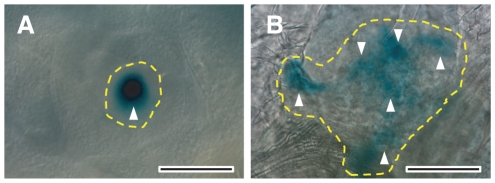
Expression patterns of the *pWUS::GUS* reporter construct. Top view of the SAM in wild type L*er* (A) and *wus-1* (B) plants. Wild-type plants show strong β-glucuronidase (GUS) activity as a single spot at the center of the SAM (A, arrowhead). In contrast, *wus-1* plants have an enlarged SAM with multiple foci of expression at very weak levels (B, arrowheads). Ten-day-old seedlings were stained with GUS overnight. The broken lines indicate the extent of the SAM. Scale bars, 10 µm.

It is evident that development of the wild type is morphologically related to the homeostasis pattern because the both keep a constant SAM size despite active cell division. In order to prove this relationship, we examined the expression pattern of a *pWUS::GUS* reporter that reflected the activity of the activator. As has been reported in many studies [19]–[26], strong expression of the reporter is detected as a single spot at the center of the SAM (Fig. 5A). This finding confirms that the wild-type SAM of *A. thaliana* corresponds to the homeostasis pattern of our model (Fig. 4C).

#### (II) SAM size

SAM size in the homeostasis pattern can be expanded by increasing 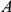 or decreasing 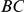 (Fig. 4B). It is generally believed that the SAM size in plants is controlled by two separate effects: first, the CZ restricts its own domain by preventing transition of PZ cells into the CZ; in addition, the CZ restricts overall SAM size by preventing differentiation of PZ cells into OZ [11], [30]. The former effect is obviously derived from the property that Turing pattern has its intrinsic spatial scale (Methods S1) [4]. On the other hand, the latter is rather related to stem cell containment, namely, PZ induction. The cooperation of the two effects determines overall SAM size.

#### (III) *wus* mutant

The model predicts that a decrease in parameter 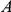 will change the wild-type homeostasis pattern to the fluctuation pattern or SAM breakdown (Fig. 4C). This prediction is supported by the similar morphological features of the *wus* mutant and the fluctuation pattern, namely, an enlarged SAM and secondary meristems initiated ectopically across the SAM (Fig. 3E) [17]. Furthermore, because expression pattern of *WUS* in *wus* mutant has not been investigated in detail, we examined in a null allele *wus-1*. We found that the expression pattern of a *pWUS::GUS* reporter in the *wus-1* mutant showed a patchy pattern at very weak levels compared to the wild type (Fig. 5B). These morphological and expressional similarities confirm the relationship between the *wus* mutant and the fluctuation pattern.

#### (IV) *stip* mutant

Mutation of *STIP* (also known as *WOX9*), which encodes a WUS homolog, produces a phenotype that is similar to but more severe than strong *wus* mutants [36]. That is, secondary shoots are never formed due to failure of growth of the vegetative SAM in the *stip* mutant. In addition, the SAM of *stip* lacks *WUS* expression. This indicates that a drastic reduction in the positive feedback causes the elimination of *WUS* expression and subsequent SAM breakdown in *stip*.

#### (V) *WUS* overexpression

**Figure 6:**
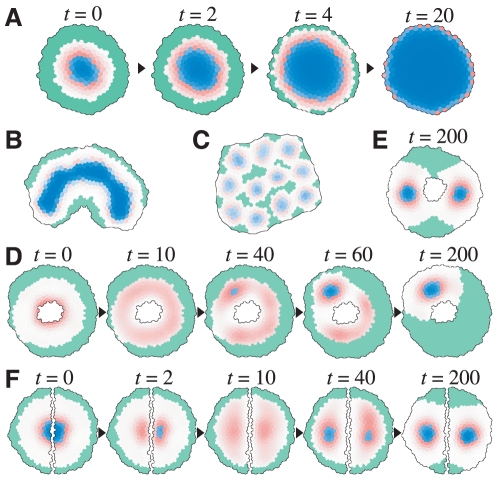
Numerical simulations for experiments affecting SAM patterning. (A) Conditional knockdown of *CLV3* causes a gradual expansion of the CZ area. (B–C) Ectopic expression of *pCLV1::WUS* (B) or *pCLV1::CLV3* (C) causes *clv*-like and *wus*-like SAM morphologies, respectively. (D–E) CZ spots reform after ablation of the CZ cells. CZ foci reform as either a single (D) or two spots (E). (F) As a result of the incision through the meristem center, the two halves reorganize into two new meristems. See also Fig. S2A (B), S2B (C), and Movies S12, S13, S14, S15 (A, D, E and F, respectively). Blue and red indicate the activator concentration in the CZ and PZ, respectively. The green area indicates the OZ.

Our model predicts that an intensified positive feedback will lead to the dichotomous or fasciation pattern (Fig. 4C). An enlarged fasciated SAM, similar to that of the *clv* mutant, is caused by strong ectopic expression of *WUS* under the *CLV1* promoter in the OC and the surrounding region [19], [23]. This morphological defect can be generated by numerical simulations using similar conditions (Figs. 6B and Fig. S7A).

#### (VI) Cytokinin effect

The plant hormone cytokinin stimulates the positive feedback pathway involving *WUS*, and thereby causes expansion of the *WUS* expression domain [26]. Knockdown of the *ARR7* and *ARR15* genes, negative regulators of cytokinin signaling, causes both increase of *WUS* expression and enlarged meristems [37]. On the other hand, SAM size is diminished by decreasing cytokinin levels [38], [39] and by defects in its signal transduction [40], [41]. In addition, *WUS* expression is also reduced by the defect of AHK2, a cytokinin receptor [26]. These experimental results are consistent with the outcome of changing parameter 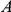 in the model (Fig. 4C). The relatively weak effects of cytokinin compared to those of *wus* mutation suggest that cytokinin has a limited involvement in the feedback regulation.

#### (VII) *clv* mutants

Defects in *clv* cause morphological abnormalities such as enlarged, fasciated, or bifurcated SAMs [14]–[16]. In addition, these structural changes are correlated strongly with the expression pattern of *WUS*. That is, *WUS* expression is expanded in enlarged SAMs and is elongated in fasciated SAMs [19], [20], [26]. These results also agree with the predictions of the model as *CLV* defects cause a reduction in parameter 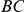 (Fig. 4C).

#### (VIII) *CLV3* knockdown

The conditional knockdown of *CLV3* results in a gradual expansion of the *pCLV3::GFP* expression area [30]. This observation is consistent with the expectations of our model (Fig. 6A and Movie S12).

#### (IX) *CLV3* overexpression

Reinforcement of the negative feedback is expected to produce a diminished homeostatic SAM, or the fluctuation pattern, or SAM breakdown (Fig. 4C). The introduction of multiple copies of *CLV3*, under its own promoter, reduces both the *WUS*-expressing domain and SAM size [23]. The relatively weak effect in this case may be due to buffering by the WUS-CLV system [23]. In addition, ectopic expression of *CLV3* under the *CLV1* promoter resulted in a *wus*-like SAM, which is closely associated with the fluctuation pattern [23]. This change in the SAM can also be produced by numerical simulations under similar conditions (Figs. 6C and Fig. S7B). Moreover, the SAM of many *p35S::CLV3* transgenic plants ceases to initiate organs after the emergence of the first leaves [20]. This is due to strong inhibition against the activator that precludes pattern formation, resulting in SAM breakdown.

#### (X) *pt* mutant

The mutant defective in *PT* (also known as *AMP1*, *COP2*, and *HPT*) forms an enlarged SAM with discrete spots of *CLV3* expression and a subsequent excess of shoots [42], [43]. These results strongly indicate that *pt* is related to the multiplication pattern in the model.

#### (XI) Meristem reorganization

In tomato, the CZ can be regenerated following laser ablation of CZ cells [44]. Our model can also produce this regeneration process (Fig. 6DE and Movies S13, S14). After CZ ablation, the activator is transiently induced in a ring-shaped region of the PZ (Fig. 6D, *t* = 10). Then, the high activator region is gradually restricted to a few spots (Fig. 6D, *t* = 40), each of which develops a stable CZ spot if the activator level exceeds the threshold for CZ (Fig. 6D, *t* = 100). This modeled regeneration process is similar to that observed in the ablation experiments [44].

In addition, the incision through the meristem center by laser ablation causes reorganization into two new meristems in tomato [45]. This experimental observation is also consistent with a model prediction (Fig. 6F and Movie S15). That is, after the incision, the activator expression is transiently reduced and dispersed (Fig. 6F, *t* = 10), but is then gradually reorganized at the center of each meristem half (Fig. 6F, *t* = 40). Finally, stable CZ spots are regenerated (Fig. 6F, *t* = 200).

## Discussion

**Figure 7:**
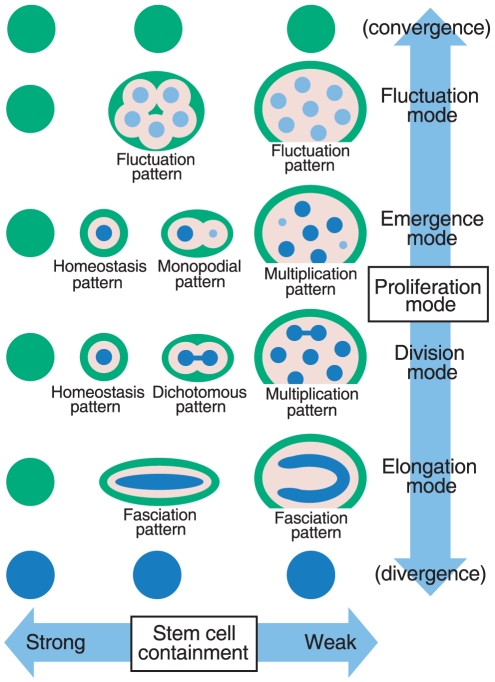
Model for SAM patterning. SAM pattern essentially depends on the proliferation mode and stem cell containment strength. The proliferation mode can be sub-divided into four groups, depending on the molecular dynamics regulating the SAM, such as WUS-CLV dynamics of *A. thaliana*. On the other hand, stem cell containment is associated with the spatial restriction of the dynamics. The blue, red and green areas indicate the CZ, PZ and OZ, respectively.

We show here that SAM patterning is essentially governed by only two parameters: the proliferation mode and stem cell containment (Fig. 7). The proliferation mode is defined by the dynamics of a molecular network, such as WUS-CLV interaction in *A. thaliana*. We also show that the proliferation mode has only four groups, and this is a common property of Turing systems [4], [31], [32]. Accordingly, the summary of our results in Fig. 7 is applicable not only to *A. thaliana* but also to all other plant species. However, since the dynamics of each regulatory network may favor particular proliferation modes, it is likely that each plant species also show preferred patterns. On the other hand, stem cell containment is achieved through a spatial restriction mechanism. Under conditions of overly strong containment, a plant will die because of SAM breakdown; however, with weak containment, the plant loses control over the SAM resulting in excessive shoots. By contrast, under intermediate containment conditions, a plant can control the cell populations in the SAM. It is likely that this homeostasis pattern is present in most plant species including *A. thaliana*. This result also provides an insight into why and how most plant species have a main shoot axis with a constant diameter.

The branching of plant shoots is classified into two types: dichotomous or monopodial. Dichotomous branching, as is observed in *Psilotum*, appears to be the equivalent of the dichotomous pattern in our model. In contrast, it is likely that the lateral branches observed in many plant species are not associated with the monopodial pattern but rather are controlled by the distinct dynamics of auxin and its carrier, PIN1, because shoots in the *pin1* mutant of *A. thaliana* elongate normally but fail to generate lateral branches [46]. Our model also provides an insight into how shoot structures of plants has evolved between monopodial shoot axis and dichotomous shoot branching.

For extending the model of this article to explain the pattern of a three-dimensional shoot, it is first required to introduce a three-dimensional cell network system that is capable of cell division. Furthermore, in order to generate the correct three-dimensional pattern of WUS-CLV expression, it seems to need spatial restrictions according to cell layer. As described in the above, spatial expressions of *WUS* and *CLV3* are strongly limited to the outermost cell layers and the underlying layers, respectively. It is likely that these spatial restrictions are not regulated by WUS-CLV dynamics itself but rather depend on an unknown upstream signaling pathway associated with cell layer differentiation. Therefore, these expressional limitations according to cell layer are required for simulating a three-dimensional meristem.

Over half a century ago, Turing first proposed the reaction-diffusion mechanism as the basis for self-organization and pattern formation in biological systems [1]. In several developmental events of animals, candidate molecules that play a central role in pattern formation by the reaction-diffusion mechanism have been proposed [6], [7]. However, it would be difficult to demonstrate reaction-diffusion activity in these cases, because morphogenetic processes in most of them are irreversible and experimental perturbations may be lethal. By contrast, the SAM of plants repetitively produces new organs throughout the lifespan. In order to demonstrate the reaction-diffusion pattern in living systems, it is thought that two lines of evidence are required [6], [7]. One is the identification of elements of interactive networks that fulfill the criteria of short-range positive feedback and long-range negative feedback. By a number of experimental studies, WUS-CLV dynamics clearly satisfies the criteria in the SAM pattern formation [10]–[13]. The other requirement is to show that a reaction-diffusion wave exists, that is, we need to identify dynamic properties of the reaction-diffusion pattern that is predicted by the computer simulation. In the case of the SAM, results of earlier studies suggest that WUS-CLV dynamics satisfies this requirement [27]–[29]. Furthermore, the findings of this article strongly reinforce this argument. Accordingly, it appears that WUS-CLV dynamics fulfills the requirement for demonstrating the reaction-diffusion pattern in the SAM. We thus conclude that the reaction-diffusion mechanism is probably indispensable for the SAM development of plants.

## Materials and Methods

### GUS Staining Analysis

The *pWUS::GUS* reporter line [47] was crossed with *wus-1*/+ heterozygous plants to produce *pWUS::GUS*+ *wus-1*/+ F_1_ plants. *pWUS::GUS* expression was analyzed in *wus-1* homozygotes in the F_2_ generation. β-glucuronidase (GUS) staining of whole mount SAMs was performed largely as previously described [19], except for use of 10 mM potassium-ferricyanide as the staining buffer. Samples were cleared in 70% ethanol and mounted in chloral hydrate. The expression pattern of *pWUS::GUS* was analyzed using a Zeiss AxioPlan2 microscope.

### Numerical Calculations

The numerical calculations were implemented in C, and the cell network dynamics and reaction-diffusion dynamics were integrated using the Euler method. The graphics of cell networks was made in Mathematica ver.4.2 (Wolfram Research Inc.).

The numerical simulations were performed by a repeated sequence of all or subsets of the four steps: cell network dynamics, reaction-diffusion dynamics, cell removal, and cell division (Fig. 1F). In the steps of the cell network dynamics and reaction-diffusion dynamics, numerical calculations were carried out until an almost steady state. That is, the step of the cell network dynamics was carried out with the total time 10.0 and the time step 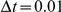, while the step of the reaction-diffusion dynamics was done with the total time 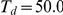 and the time step 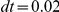.

As the shoot lengthens, cells become increasingly distant to the SAM with downward move. To accommodate this fact, cells leave the cell networks after becoming sufficiently distant from the SAM. That is, in the cell removal step, we remove cells from the cell network if 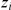 is lower than a threshold level, 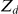, that is smaller than 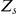. In the cell division step, the largest cell divides in a random direction with the exception of Fig S5.

The initial value of variables was given as their equilibrium with a random fluctuation of 1.0%, and numerical simulations of the reaction-diffusion dynamics were imposed on the boundary condition of zero flux. Steps and parameter values used in each numerical simulation are summarized in Table S1.

### Numerical Condition in Figure S1

A two-layered lattice was obtained by the periclinal division of a single-layered lattice with 1,000 cells generated by cell division from an initial lattice with four cells. We examined three spatial restriction conditions by varying activator and inhibitor syntheses. In Fig. S1A, the activator and inhibitor are synthesized in all the cells using Equations (1) and (2a). By contrast, in Fig. S1B, they are synthesized separately in the upper and lower layers with the equations of
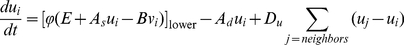
(6a)

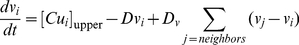
(6b)with
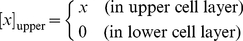
(7a)

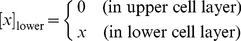
(7b)In Fig. S1C and D, a diffusible molecule 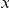 was introduced into the dynamics of Equations (6); this molecule is active in the signal transduction pathway from the activator to the inhibitor. Then the set of differential equations used is given by
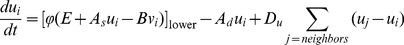
(8a)

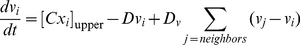
(8b)

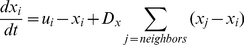
(8c)where 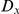 is a constant. Thus 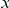 is induced by the activator, diffuses with the diffusion coefficient 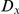, and stimulates the inhibitor.

### Numerical condition in Figures 2 and S2, S3, S4

Cell number increased from 10 to 1,000 cells without cell removal. We used Equations (1) and (2a) in Fig. 2A and S2, Equations (3) in Fig. S3, and Equations (1) and (2b) in Fig. S4. In Fig. 2B–E, we used the following modified version from Equations (1);
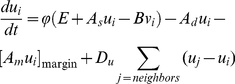
(9a)

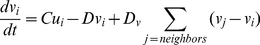
(9b)with
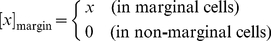
(10)where 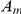 is a positive constant. Thus the activator has a higher rate of degradation in marginal cells than in non-marginal cells, and this condition prevents CZ spots from migrateing to the edge of the cell network.

### Numerical Condition in Figures 3, 4, and S6

We used the following form modified from Equations (4);
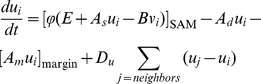
(11a)

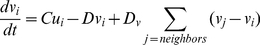
(11b)

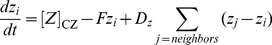
(11c)where positive constant 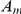 is introduced in order to prevent CZ spots from migrating to the edge of the cell network as in the case of Equations (9). Pattern development by a Turing system requires a minimal area size that depends on dynamics parameters [4]. Accordingly, in the previous step, the activator is synthesized in all cells and cell number is increased without cell removal, until a CZ spot emerges and is maintained stably in the center of the SAM. Then, the dynamics expressed by Equations (11) was applied until the cell population reach 1000 cells together with removal cells. The resultant SAM patterns were subjected to pattern classification. The dominant pattern for each set of parameter conditions was determined after at least ten independent numerical simulations.

### Numerical Condition in Figure S5

Numerical simulations were carried out as in Fig. 3F except for the cell division step. Cell division occurs in the cell with the largest value of multiplying its cell volume by a constant factor; 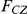 in CZ, 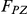 in PZ, or 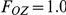 in OZ. Therefore, the activity of cell division becomes high according to the increase in this factor. The dominant pattern for each set of parameter conditions was determined after at least ten independent numerical simulations.

### Numerical condition in Figure 6A

The homeostasis pattern was initially generated as in Fig. 3F, and then the synthesis of the inhibitor was interrupted by reducing parameter *C* to zero.

### Numerical Condition in Figures 6BC and S7

*CLV1* is strongly expressed in the OC and its surrounding region; however, the regulatory mechanism of this expression pattern has not yet been clarified. We, therefore, assumed a diffusible signal molecule (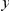) that is induced by the activator, and diffuses at the rate 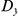 to stimulate the *CLV1* promoter with an intensity of 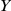 (see Fig. S7). Consequently, sets of equations under the condition overexpressed by the *CLV1* promoter are described by
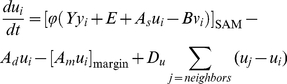
(12a)

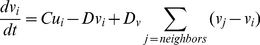
(12b)

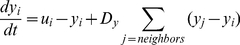
(12c)

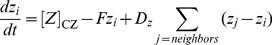
(12d)for *pCLV1::WUS* (Figs. 6B and S7A) and
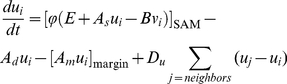
(13a)

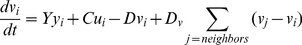
(13b)

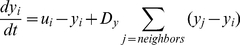
(13c)

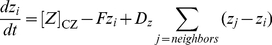
(13d)for *pCLV1::CLV3* (Figs. 6C and S7B). The dominant pattern for each set of parameter conditions was determined after at least ten independent numerical simulations.

### Numerical Condition in Figure 6D–F

After SAM in the homeostasis state was initially generated as in Fig. 2F, the CZ cells (Fig. 6DE) or a line of cells through the meristem center (Fig 6F) was removed from the cell network. Then the calculations were carried out in the same way as before the cell ablation.

## Supporting Information

Figure S1 **Effect of expressional separation between the activator and inhibitor in a two-layered cell network.** (A) Stable patterns are developed without any spatial restrictions under the Turing condition (inside the dashed lines, see Methods S1). (B) However, stable patterns are completely eliminated by introducing expressional separation in which the activator and inhibitor are synthesized only in the lower and upper cell layers, respectively. (C) In contrast, stable patterns are restored by introducing another diffusible signal molecule (*x*) into the signaling pathway from the activator to the inhibitor. (D) Pattern restoration requires that the diffusion coefficient of *x* (*D_x_*) is sufficiently larger than that of the activator (*D_u_* = 0.25). Filled circles and crosses indicate stable patterns and no stable patterns, respectively. In A–C, the area enclosed by the dashed lines indicates the Turing condition of Equations (3) (see Methods S1).
(EPS)

Figure S2
**Effect of** ***C*** **on the proliferation mode.** Parameter 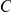 has the same effect as 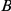 on the proliferation mode (compare with Fig. 2A).(EPS)

Figure S3 **Effect of a simplified dynamics on the proliferation mode.** The simplified activator-inhibitor dynamics described by Equations (3) shows a similar result to that by Equations (1) (compare with Fig. 2A).
(EPS)

Figure S4
**Effect of the upper limitation of the activator on the proliferation mode.** (A) Patterns are responsive to the ratio of distances from the equilibrium of the activator (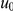) to the upper limitation (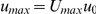) and lower limitation (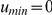). (B–F) Pattern evolutions of the stripe mode (B, double circles), reverse fluctuation mode (C, open diamonds), reverse emergence mode (D, open triangles), reverse division mode (E, open squares), and reverse elongation mode (F, open circles). Filled diamonds, filled triangles, filled squares, and filled circles indicate the fluctuation mode, emergence mode, division mode, and elongation mode, respectively.(EPS)

Figure S5
**Effect of relative frequency of cell division between CZ, PZ, and OZ on the homeostasis pattern formation.** Cell division frequency is varied by changing parameters 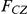 and 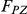 (for detail see Materials and Methods). Blue, magenta, and green in bar graphs indicate relative frequency of cell division in CZ, PZ, and OZ, respectively. Open circles and open squares indicate the homeostasis pattern and dichotomous pattern, respectively.(EPS)

Figure S6
**Effect of** ***C*** **on SAM pattern formation.** 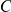 has the same effect as 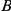 on the SAM pattern formation (compare with Fig. 4A) as in the case of the proliferation mode.(EPS)

Figure S7
**Effect of ectopic expression of** ***WUS*** **or** ***CLV3*** **driven by the** ***CLV1*** **promoter.** We assumed a signal molecule (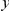) that is induced by the activator, diffuses with the diffusion coefficient 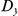, and stimulates the *CLV1* promoter with the strength 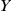. (A) Ectopic expression of *pCLV1::WUS* induces morphological alteration from the wild-type homeostasis pattern (open circles) to a *clv*-like dichotomous (open squares) or fasciation (filled circles) pattern. (B) On the other hand, ectopic expression of *pCLV1::CLV3* causes the fluctuation pattern (filled diamonds) that is similar to the phenotype of the *wus* mutant. Crosses indicate conditions where no patterns are generated, and filled square indicates the multiplication pattern by the division mode proliferation.(EPS)

Table S1 **The steps and parameter values used in the numerical simulations.**
(XLS)

Methods S1 **Theoretical background of the reaction-diffusion system and numerical condition of the cell network dynamics.**
(DOC)

Movie S1 **Pattern evolution of the fluctuation mode in** **Fig. 2B** **.**
(MOV)

Movie S2 **Pattern evolution of the emergence mode in** **Fig. 2C** **.**
(MOV)

Movie S3 **Pattern evolution of the division mode in** **Fig. 2D** **.**
(MOV)

Movie S4 **Pattern evolution of the elongation mode in** **Fig. 2E** **.**
(MOV)

Movie S5 **Time evolution of the fasciation pattern in** **Fig. 3B** **.**
(MOV)

Movie S6 **Time evolution of the multiplication pattern by the division mode in** **Fig. 3C** **.**
(MOV)

Movie S7 **Time evolution of the multiplication pattern by the emergence mode in** **Fig. 3D** **.**
(MOV)

Movie S8 **Time evolution of the fluctuation pattern in** **Fig. 3E** **.**
(MOV)

Movie S9 **Time evolution of the homeostasis pattern in** **Fig. 3F** **.**
(MOV)

Movie S10 **Time evolution of the dichotomous pattern in** **Fig. 3G** **.**
(MOV)

Movie S11 **Time evolution of the monopodial pattern in** **Fig. 3H** **.**
(MOV)

Movie S12 **CZ expansion caused by the** ***CLV3*** **knockdown in** **Fig. 6A** **.**
(MOV)

Movie S13 **Regeneration of a single CZ spot after ablation of the CZ cells in** **Fig. 6D** **.**
(MOV)

Movie S14 **Regeneration of two CZ spots after ablation of the CZ cells in** **Fig. 6E** **.**
(MOV)

Movie S15 **Reorganization of CZ spots after incision through the meristem center in** **Fig. 6F** **.**
(MOV)
