# Supplementary material for: Reaction-Diffusion Pattern in Shoot Apical Meristem of Plants
Source: PLoS One. 2011 Mar 29;6(3):e18243. doi: 10.1371/journal.pone.0018243 (PMC3066213; doi:10.1371/journal.pone.0018243)
Supplement: Methods S1 — Theoretical background of the reaction-diffusion system and numerical condition of the cell network dynamics. (DOC) [file pone.0018243.s009.doc]

“Reaction-Diffusion Pattern in Shoot Apical Meristem of Plants”

**by
Hironori Fujita, Koichi Toyokura, Kiyotaka Okada, an
 Masayoshi Kawaguchi**

**Supporting
Method
 S1**

**Theoretical Background of th**
**Reaction-Diffusion System**

We briefly describe here
the theoretical background of the
reaction-diffusion system for better understanding of the model presented in this article. A reaction-diffusion system involves diffusible factors interacting with each other and can develop stable spatial patterns from an almost homogeneous state by self-organization [S1]

In the case of two diffusible factors denoted by *u* and *v*, we can express
the dynamics of interaction by the
following partial differential equations:

(S1a)

(S1b)

where *D_u_* and *D_v_* are the
diffusion coefficients of *u* and *v*, respectively.
Nex
, we consider the condition that the equilibrium

 of Equations (S1
 is stable without space but is destabilized by introducing space. This condition is summarized in the following set of inequations:

(S2a)

(S2b

(S2c

(S2d

where

 is the partial derivative of *f* by *u* to be evaluated at the equilibrium and so on [S1
.
This requirement is referred to as the
“Turing condition”, and the model satisfying this condition is called the
“Turing system”.
One of the most famous Turing systems is the activator-inhibitor system that satisfies

(S3

In this cas
, *u* and *v* are conventionally terme
 the activator and inhibitor, respectively [S1–
3
.
Now
 consider the following reaction terms that are one of the simplest descriptions of
the activator-inhibitor system:

(S4a

(S4b)

where

,

,

,

,
and

 are positive constants.
The
Turing condition in this case is then described
by the
inequations:

(S5a)

(S5b)

(S5c)

(S5d)

This parameter condition was used in Fig. S1
–C (dashed lines
.
When the
Turing condition is satisfied, the wave number *k* that can grow with time is consequently described by

(S6

Furthermore, the wave number *k_max_* with the fastest growth rate is given by

(S7

This indicates that patterns developed by the
Turing system have an intrinsic spatial scale that is e
large
 by increasing

 and

 or by decreasing

 and


 This parameter dependence is consistent with the result show
 in Fig. 4B.

Now let us consider the dynamics of Equations (S1) and (S4) in one spatial dimension described by

(S8a)

(S8b)

After the substitutions of

,

,

, and


we obtain
a nondimensionalized form expressed by

(S9a)

(S9b)

where

,

, and

 are constants. This transformation shows that

 and

 have the same effect on the dynamics

This is consistent with numerical results in Fi

. 2 and S2 and in Fi
s.4A and S6
 In addition, this nondimensionalization also indicates that th

feedback
strength
 of the positive and negative regulatory loops correspond to

 and

, respecively. Furthermore, in the case that

 is a constant, these strengths are qualitatively associated with

 and

, respecivel
 (see Fig. 1D
.

**Cell
etwork.**

We used a cell network system similar to that of Prusinkiewicz and Lindenmayer [S4
. Polygonal cells are tightly arranged in two-dimensional space and thus are separated from each other by a straight cell wall.
The *i*-th cell has an outward turgor pressure

) in inverse proportion to its volume

 that can be expressed by

, where

 is a constant.
In addition, it is assumed that each cell wall side behaves like a spring in which the force acting on a vertex is given by the Hooke’s law formula

, where

,

, and

 are the spring constant, the current spring length, and spring rest length, respectively.

,

 and

 are always set as 1.0, 0.1 and 0.3, respectively. Accordingly, the total force

 acting on the *i*-th vertex is the summation of turgor pressures by the surrounding cells and of elastic forces by the connecting cell walls. We also assumed that inertial forces acting on the cellular network could be neglected. Therefore, the position of the vertex

) within a small time interval moves in the direction of and is proportional to the total force

:


 Cellular position was calculated to the equilibrium state by 1,000 iterations with a time step of

.

**References**

S1
 Murray JD (2003
 Mathematical Biology II: Spatial Models and Biomedical Applications
 Berlin: Springe
.

S2. Meinhardt H (1982) Models of Biological Pattern Formation. London: Academic Press.

S3. Meinhardt H (1995) Algorithmic Beauty of Sea Shells. Berlin: Springer-Verlag.

S4. Prusinkiewicz P, Lindenmayer A (1990) The Algorithmic Beauty of Plants. New York: Springer.
